# ImmuMethy, a database of DNA methylation plasticity at a single cytosine resolution in human blood and immune cells

**DOI:** 10.1093/database/baac020

**Published:** 2022-04-01

**Authors:** Huiying Qi, Shibin Song, Pingzhang Wang

**Affiliations:** Department of Health Informatics and Management, Peking University Health Science Center, No. 38 Xueyuan Road, Beijing 100191, China; Information Technology Center, Peking University Health Science Center, No. 38 Xueyuan Road, Beijing 100191, China; Department of Immunology, NHC Key Laboratory of Medical Immunology, School of Basic Medical Sciences, Peking University Health Science Center, No. 38 Xueyuan Road, Beijing 100191, China; Peking University Center for Human Disease Genomics, No. 38 Xueyuan Road, Beijing 100191, China

## Abstract

Differential DNA methylation is a feature of numerous physiological and pathological processes. However, the extent to which single-base cytosine methylation modifies cellular responses to various stimuli has not been well characterized. In this study, we carried out a systematic analysis of methylome data derived from human blood and immune cells and constructed the ImmuMethy database. ImmuMethy allows interrogation of DNA methylation plasticity (MPL) at the single cytosine level. MPL, which refers to the variability of DNA methylation, is quantitatively measured in multiple ways, such as quartiles and standard deviations. ImmuMethy comprises over 36 000 samples from the Human Methylation450 and MethylationEPIC BeadChips platforms and provides multiple applications, such as an overview of methylation status and plasticity, differential methylation analysis, identification of methylation markers and sample stratification. An analysis of all datasets revealed that DNA methylation is generally stable, with minimal changes in beta values. This further supports the characteristics of DNA methylation homeostasis. Based on the beta value distribution, we identified three types of methylation sites: methylation tendency sites, unmethylation tendency sites and dual tendency or nonbiased methylation sites. These sites represent different methylation tendentiousness of DNA methylation across samples. The occurrence of multiple methylation tendencies in a site means split methylation, which generally corresponds to high MPL. Inverted methylation tendencies from methylation tendency sites to unmethylation tendency sites, or vice versa, represent strong differential methylation in response to conditions. All these sites can be identified in ImmuMethy, making it a useful tool for omics-based data-driven knowledge discovery.

**Database URL**: http://immudb.bjmu.edu.cn/immumethy/

## Introduction

DNA methylation is a major epigenetic modification involving the addition of a methyl group to the C5 position of cytosine to form 5-methylcytosine (5mC). In mammals, 5mC is predominantly seen at cytosine-phosphate-guanosine (CpG) dinucleotides, although methylation events can also occur in non-CpG contexts (1). In most somatic tissues, the majority (>80%) of CpG dinucleotides found in the genome are methylated, with the exception of CpG islands (CGIs) and other gene regulatory sequences that show reduced 5mC levels (1). DNA methylation is functionally involved in many processes, such as genomic imprinting, X chromosome inactivation, silencing of repetitive DNA elements and regulation of tissue-specific gene expression (2).

High-throughput technologies, such as microarrays and next-generation sequencing (NGS), have been widely used to identify differentially methylated sites in various contexts, including normal human development, aging, tumorigenesis, autoimmune disorders, neurological disorders, immunodeficiency, species evolution, environmental adaptation and genetic and epigenetic diseases. As a result, there is an abundance of epigenome-wide DNA methylome data available. Therefore, the systematic collection, integration and utilization of these data will undoubtedly contribute to the field, enabling an overview of gene methylation levels under various conditions, identification of the underlying regular methylation patterns and emerging new biological insights into methylation roles. In addition, re-evaluation of methylation biomarkers, such as cell type-specific and disease-associated methylation biomarkers, is also an important task for omic big data usage. Database construction is an excellent way to share large datasets and promote data usage in further experimental research. Currently, several methylation databases have been built, including MethDB (3, 4), PubMeth (5), MethyCancer (6), MENT (7) and MethHC (8). However, most existing methylation databases are specifically related to cancer.

Blood and immune cells are often used in methylation studies due to the ease of sample collection, resulting in abundant methylome data for these cells. Although there are some databases, such as epigenome BLUEPRINT (9, 10) and MethBase (11), which contain immune cell methylome data, their datasets are from NGS. In addition, there are still very limited samples and experimental conditions for the same cell types in these databases. In this study, we present a new database, ImmuMethy (http://immudb.bjmu.edu.cn/immumethy/), which aims to collate available methylome data for human blood and immune cells. Integration of mouse methylation data will be considered in subsequent versions of ImmuMethy once sufficient data have been compiled. It currently comprises over 36 000 samples from both the Human Methylation450 (or 450k) and MethylationEPIC (or 850k) BeadChips platforms.

ImmuMethy provides data for DNA methylation at single cytosine bases. It can provide information on DNA methylation levels in human blood and immune cells derived from different disease states and tissue sources. ImmuMethy emphasizes a global view of methylation profiles due to the large sample size. Samples are categorized into different study datasets based on sample source, cell type and disease state. Therefore, the database can illustrate methylation profiles and plasticity and permits examination of the dynamic changes in DNA methylation that occur under various experimental conditions. In addition, further analyses, such as differential methylation profiles, methylation marker evaluation and tracing experimental conditions based on the methylation level, can also be carried out using the database.

## Materials and methods

### Datasets and processing

All data related to the Infinium Human Methylation450 (450k) and MethylationEPIC (850k) BeadChips, as well as the platform annotation files, were downloaded from the GEO (12) and ArrayExpress (13) databases. Platform annotations include basic probe information, such as gene symbols, chromosomes, single nucleotide polymorphisms (SNPs), gene region features [including untranslated regions (UTRs), gene bodies, transcription start sites (TSSs) and the first exons] and position relationships with canonical CGIs. For example, the shores are denoted as being 0–2 kb from CGIs, whereas shelves are 2–4 kb from CGIs. SNP information related to array probes was directly downloaded from the official websites https://support.illumina.com/array/array_kits/infinium_humanmethylation450_beadchip_kit/downloads.html and https://support.illumina.com/array/array_kits/infinium-methylationepic-beadchip-kit/downloads.html. Chromosomal coordinates of methylation sites were converted from the human genome hg19 to hg38 assembly using the liftOver program (https://genome.ucsc.edu/cgi-bin/hgLiftOver). The 450k array interrogates over 450 000 methylation sites (predominantly CpG dinucleotides) across the human genome at a single-nucleotide resolution, covering 99% of reference sequence (RefSeq) genes and comprises the most abundant sample type across all current methylation platforms The 850k array covers the majority of the sites from the 450k array but adds 333 265 CpGs located in enhancer regions; therefore, it interrogates a total of over 850 000 CpG sites for methylation status assessment (14).

Sample information was exported to an Excel file for further manual checks, and datasets from human blood and primary immune cells were selected. The raw data in the IDAT file format were downloaded and uniformly processed using the R (http://www.r-project.org/) package “minfi” (15) to produce the uniformly processed beta values used in the study. The beta value is the ratio of the methylated probe signal intensity to the overall intensity, that is, beta value = Meth/(Meth + Unmeth), where “Meth” and “Unmeth” indicate methylated and unmethylated probe signals, respectively. The beta value ranges between 0 and 1, with 0 being unmethylated and 1 being fully methylated. A beta value of 0.5 indicates balanced signal levels between the methylated and unmethylated probes.

In this study, beta values were directly calculated with the function “getBeta” with a default offset of 100, which was chosen to avoid dividing by small values. A detection *P*-value was returned for every genomic position using the function “detectionP.” Positions with nonsignificant *P-*values larger than 0.01 indicate that there is no difference between the DNA signal and the background signal estimated using negative control positions. To adjust for Infinium I and Infinium II probe design, the Subset-quantile Within Array Normalization (SWAN) algorithm (16) was used for beta value normalization within arrays to reduce the technical variation due to the probe types while maintaining the important biological differences.

In some methylation-related studies, the data submitter did not provide the IDAT format raw files during data submission to GEO or ArrayExpress but rather a single, large beta value matrix of all samples. These preprocessed or submitter processed beta values from either normalized or nonnormalized data cannot be directly queried because of their complex data structure. We parsed these matrices and integrated all of the data for user queries (see section “Discussion”). During quality control, samples comprising abnormal beta values less than zero, larger than 1 or without a beta value were removed.

### Analysis of beta value distribution and methylation plasticity

For the normalized beta values in each study dataset, the “fivenum” function in the R (http://www.r-project.org/) software environment was used to calculate the minimum (Min.), first quartile (25th percentile, first Qu, Q1), median, third quartile (75th percentile, third Qu, Q3) and maximum (Max.) values. To systematically estimate the variability in DNA methylation or methylation plasticity (MPL), we calculated the standard deviation and quantile range to quantify the amount of dispersion or spread of beta values. In this study, we used two types of quantile ranges: “quantile range (Q75–Q25)” or the interquartile range (IQR), which is the difference between the 75th and 25th quantiles of the beta values, and “quantile range (Q95–Q5),” which is the difference between the 95th and 5th quantiles of the beta values. Quantile ranges are robust to outliers and allow enough samples to support methylation variability effectively and avoid potential negative biases due to experimental and probe design. The relationships among the three measures were compared by using the Pearson correlation method.

### Graph plotting of methylation profile

For graph plotting of the methylation profile, the normalized beta values of each methylation site in a specific study dataset were first rounded to two decimal points. This will make the beta values ascribed to 101 numerical numbers, and sample proportions at each number were calculated to produce a methylation profile matrix. A rounded beta value (r-beta) curve, which connects all sample ratios on the *y*-axis at the 101 rounded values on the *x*-axis, was used to graphically illustrate methylation profiles. The shape, including width and height, as well as the peak position on the *x*-axis, can indicate the overall methylation intensity and its variability.

### Homogeneity test of beta values

Duda–Hart tests were performed using the “dudahart2” function in the flexible procedures for clustering (fpc) package (17) to determine whether a set of beta values should be split into two or more clusters. If the returned result of “cluster 1” is false, the null hypothesis of homogeneity is rejected. For the CpG sites with more than 1 cluster of beta values, the “pamk” function (https://www.rdocumentation.org/packages/fpc/versions/2.2-9/topics/pamk) with default parameters was further used to determine the numbers of clusters. The function uses the most common k-medoids clustering method “pam” algorithm to perform partitioning around medoids clustering and returns a suggested number of clusters.

### Methylation tendency analysis

Methylation tendency refers to the overall tendency of a cytosine site to be methylated or unmethylated in the sample populations. Because a beta value of ∼0.5 indicates balanced signal levels between the methylated and unmethylated probes based on the formula of beta value calculation, beta value ranges of 0.5–1 (0.5 < beta value ≤ 1) and 0–0.5 (0 ≤ beta value < 0.5) reflect biased methylated (0.5–1) or unmethylated (0–0.5) probe signals, respectively. We used a chi-square goodness of fit test with the R function “chisq.test” to determine if there was a statistically significant difference in beta value distribution in the two data regions. We considered a site as a methylation tendency site (MTS) or an unmethylation tendency site (UTS) when the *P*-value was less than or equal to 0.05 if more beta values were located in the range of 0.5–1 for MTS or in the range of 0–0.5 for UTS. In contrast, a site was considered a Dual Tendency methylation Site (DTS) or nonbiased methylation site (NTS) when the *P*-value was larger than 0.05. The methylation tendency of a queried site can be clearly illustrated via a methylation profile curve when the peak will be located mainly on the right (MTS), left (UTS) and middle or both of the left and right (DTS) sides of the curve with almost equal sample proportions.

### Differential methylation analysis

For an online differential methylation analysis among methylation sites, the Mann–Whitney *U*-test (also called Wilcoxon rank-sum test) was automatically conducted in response to the user’s operation with the function “MannWhitneyUTest” (http://commons.apache.org/proper/commons-math/javadocs/api-3.0/org/apache/commons/math3/stat/inference/MannWhitneyUTest.html). For offline analysis, the R function “wilcox.test” was used for the Wilcoxon rank-sum test. The null hypothesis of the Wilcoxon test is that the two populations have the same distribution with the same median. A *P*-value less than or equal to 0.05 is generally statistically significant. However, considering the multiple comparison problem and hundreds of thousands of cytosine sites from both platforms, individual online tests with a *P*-value  < 1.00E-7 are suggested to be significant enough for differential methylation levels according to the widely used Bonferroni correction method. The formula for a Bonferroni correction is as follows:

$${{\rm{\alpha }}_{{\rm{new}}}}{\rm{ = }}{{\rm{\alpha }}_{{\rm{original}}}}{\rm{/n}}\vspace*{6pt}$$

where *α*_original_ indicates the original *α* level and *n* represents the total number of comparisons or tests being performed. In addition to a Bonferroni correction, a less stringent Benjamini and Hochberg’s false discovery rate controlling method, which takes both the total number of tests and the individual *P*-value’s rank into consideration, was also used for the differential methylation analysis. The R function “p.adjust” returns the adjusted *P*-value s with the “bonferroni” and “BH” (aka “fdr”) methods for multiple comparisons.

### Analysis of transcription factors with methylated DNA binding

Some transcription factors (TFs) recognize methylated DNA and play an important role in gene regulation. Therefore, we integrated TF binding information into ImmuMethy. Data on the interactions between methylated DNA and TFs were downloaded from the MeDReaders database (18), which curates hundreds of TFs that could bind to methylated DNA sequences. The human data are from six human cell lines, including GM12878 (human lymphoblastoid cell line by Epstein-Barr virus (EBV) transformation), H1-hESC (human embryonic stem cell line H1), HCT116 (colorectal carcinoma cell line), HepG2 (hepatocellular carcinoma cell line), IMR-90 (human diploid fibroblasts from fetal lung) and K562 (human myeloid leukemia cell line), based on whole-genome bisulfite sequencing (WGBS) and ChIP-Seq experiments (18). The exact coordinates of the predicted methylated and unmethylated cytosines with TF binding were first extracted, then the sites that were interrogated by either 450k or 850k arrays were retained for further analysis.

### Database construction

ImmuMethy was implemented based on the Client Browser/Web Server/Database Server three-tier architecture. It was built with the model–view–controller development framework. Servlet was used as a controller, with Java server page as a view component and Java Bean class as a model. The data were stored and managed by a MySQL relational database (version 8.0.17). The current version of ImmuMethy runs on an Apache Tomcat web server (version 6.0.45). It accesses the database using Java Database Connectivity.

## Results

### Data statistics

**Figure 1.**
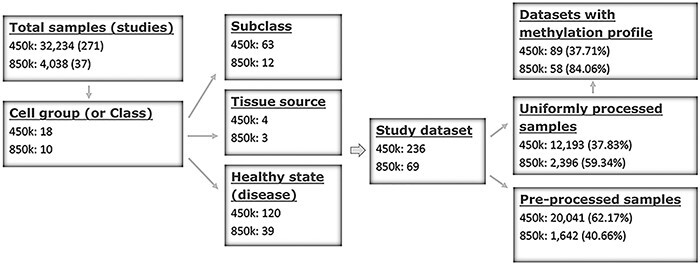
Sample stratification and statistics. (A) Sample or dataset counts are shown beside the platforms: for 450k, ∼37.83% (12 193/32 234) of all samples are uniformly processed, whereas, for 850k, ∼59.34% (2396/4038) of all samples are uniformly processed. In addition, ∼37.71% (89/236) and ∼84.06% (58/69) of all study datasets had methylation profiles. In total, ∼62.29% (147/236) and ∼15.94% (11/69) of all study datasets for the 450k and 850k platforms, respectively, were not uniformly processed, and for these study datasets, only the preprocessed beta values but not methylation profiles could be analyzed. For more details, please see Supplementary Tables S1 and S2.

ImmuMethy uses beta values to quantify methylation levels. To include as many experimental conditions as possible for the downstream DNA methylation analysis, two types of beta values are reported: the uniformly processed values, which were recalculated in this study from the raw IDAT format files, and the preprocessed beta values, which were previously determined by the data submitter for which no IDAT files were provided. ImmuMethy integrates blood and immune cell data from 308 studies with different sample tissue sources, cell types and disease states (or experimental conditions), involving 36 272 samples from the two platforms, 450k and 850k (Figure 1, Supplementary Tables S1 and S2). Each cell group (or cell class on the query web page) may comprise certain subclasses based on cell phenotypes and functions, and these subclasses are further grouped by disease or experimental condition. In ImmuMethy, sample data reported in different studies but derived from the same tissue source, cell subclass and experimental condition are grouped into a single study dataset, which is assigned a unique study dataset ID. Detailed sample stratification and metadata information are shown in Supplementary Tables S1 and S2.

For example, for the 450k platform, the CD4^+^ T cell class is separated into several subclasses, including naive CD4^+^ T cells, memory CD4^+^ T cells, central memory CD4^+^ T cells and effector memory CD4^+^ T cells. These subclasses are derived from different human peripheral blood samples representing different disease states, such as naive CD4^+^ T cells in peripheral blood derived from healthy individuals (PB_naiveCD4T_normal) or systemic lupus erythematosus (SLE) patients (PB_CD4Tnaive_SLE).

For the 450k platform, there were a total of 32 234 nonredundant samples, including 12 193 (37.83%) samples with uniformly processed beta values and 20 041 (62.17%) samples with preprocessed beta values. These samples were divided into 236 study datasets based on tissue source, cell type and disease state (Figure 1, Supplementary Tables S1 and S2). For the 850k platform, there were a total of 4038 nonredundant samples, including 2396 (59.34%) samples with uniformly processed beta values and 1642 (40.66%) samples with preprocessed beta values. These samples were divided into 69 study datasets based on tissue source, cell type and disease state (Figure 1, Supplementary Tables S1 and S2).

### Query

**Figure 2.**
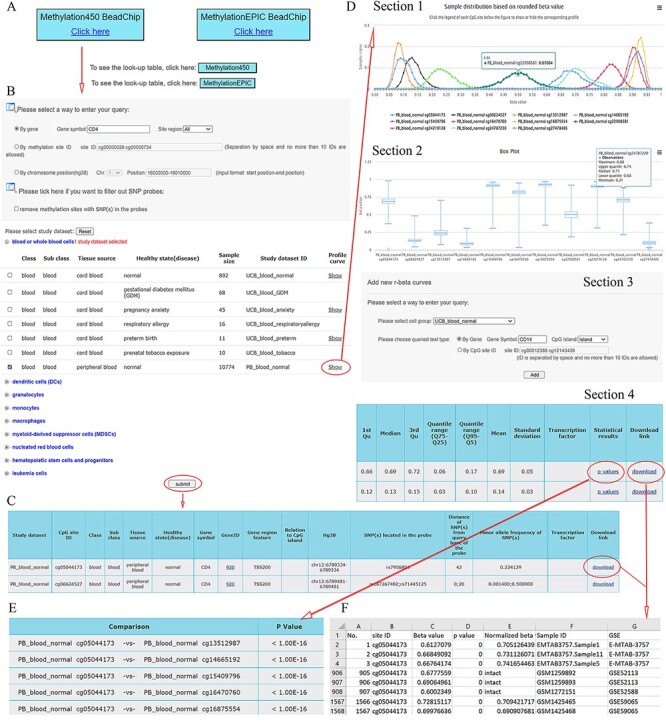
A workflow to navigate the database, using CD4 as an example. (A) The Methylation450 BeadChip platform is selected. B. The gene symbol “CD4” was used to search “All” possible methylation sites in the study dataset PB_blood_normal (normal human peripheral blood cells). The “Reset” button is used to deselect all study datasets. C. On the beta value result page, a basic description of all retrieved methylation sites is given. Beta values can be downloaded via the “download” button. D. On the methylation profile result page, four sections are included. Section 1 directly shows methylation profiles by the r-beta curve, which indicates the extent of MPL, the distributions of beta values and the overall methylation intensities. Section 2 uses box plots to show beta value distributions. Section 3 provides a curve addition function, facilitating the addition of new profiles for comparison. Section 4 shows the basic descriptions, such as quartiles, mean, standard deviation and quantile ranges of the beta values, for all retrieved sites. Beta values and results of statistical comparisons can be downloaded via the corresponding links. E. Statistical comparisons between the site in the indicated row and the sites in the other rows. A lower *P*-value indicates a greater statistical significance of the observed differential methylation levels. F. The beta value result. The meaning of the headings is shown as follows. The “p value” reflects statistical significance between the true hybridization signal and the background signal. The “intact” symbols, but not the numeric values (which represent recalculated values), in the column “Normalized beta value” indicate the beta values in the column “Beta value” represent preprocessed values that are directly from the data submitter. Sample IDs and data source study IDs are shown in the columns “Sample ID” and “GSE,” respectively. See the operation steps in the main text for more details. Web page figures are cropped to fit the page.

ImmuMethy provides a user-friendly interface to query DNA methylation at single cytosine sites. It supports gene symbols/aliases (e.g. CD4), methylation site IDs (e.g. cg00000029) and chromosome positions (e.g. 16 000 000–16 010 000 on chromosome 1) as search terms as output, both beta values and methylation profile queries are provided. Figure 2 shows the search term “CD4” as an example workflow.

Step 1. Select a platform (Human Methylation450 and MethylationEPIC BeadChips platforms). Look-up tables are provided for the two platforms provided below the click buttons (Figure 2A).

Step 2. Enter the query (Figure 2B). As an example, the gene symbol CD4 is used to search all possible cytosine sites (“All” in the pull-down menu of the site region) in the first exons, UTRs, gene bodies, CGIs, shores (0–2 kb from CGIs) and shelves (2–4 kb from CGIs). When the filter option is checked, the sites containing SNPs in their array probes will not appear in the query results.

Step 3. Select study datasets of interest (Figure 2B). Selecting the plus sign before each cell group can expand or collapse each study dataset. Multiple study datasets can be selected. Next, there are two options for how to proceed.

Option 1. Click on the submit button at the bottom of the query web page to retrieve beta values. This result page is shown in Figure 2C. There are basic descriptions of the retrieved methylation sites available on the page (also see online FAQs for detailed explanation).

Option 2. Click on the “Show” button to see the methylation profiles (Figure 2D), which are derived only from uniformly processed data. On the methylation profile result page, there are four sections. Methylation profiles are shown as r-beta curves (see section “Materials and methods” for details). Several different profiles can be visualized at once to allow comparison of the beta value distributions.

Step 4. For Option 2, click on the “p values” button to retrieve statistical comparisons between the site in the indicated row and that site in the other rows (Figure 2E). The *P*-values are directly from the Mann–Whitney *U*-test, which is automatically conducted in response to the user’s operation. The lower the *P*-value, the greater the statistical significance of the observed differential methylation levels. However, statistical comparisons are conducted only for uniformly processed data.

Step 4. Click on the “Download” button seen in Figure 2C and D (Section 4) to show detailed results, including all beta values, in an Excel format file. In the beta value result page, brief information about retrieved sites is also shown (Figure 2C). The uniformly processed and/or preprocessed beta values of all retrieved methylation sites from the query are included in Excel files with one file corresponding to one site (Figure 2F). All result files can be downloaded simultaneously via the button “Download all” at the bottom of the result page for further analysis.

Step 4. Click on the “see more” button if there are TFs predicted to bind the indicated cytosines with either high or low methylation levels. The TF–DNA interaction information, such as tissue source, predicted binding sequence, CpG site in the sequence and its methylation level, is shown. The information is directly derived from the MeDReaders database (see below for more details).

Step 5 (optional). New profiles were added to the current methylation profiles (Figure 2D, Section 3). The addition of new profiles can be performed on the profile result page to facilitate profile comparison.

### Application

ImmuMethy has methylation data for a total of 18 161 003 429 sites from over 36 000 samples, including 15 133 393 219 (83.32%) sites from the 450k platform and 3 027 610 210 (16.67%) sites from the 850k platform. In addition, there were 93 455 099 methylation profiles, with 43 210 568 (46.24%) profiles from the 450k platform divided into 89 study datasets and 50 244 531 (53.76%) profiles divided into 58 study datasets from the 850k platform (Figure 1), all of which could be searched and compared. This allows the comparison of 9.34e + 14 and 1.26e + 15 pairwise profile combinations for the 450k and 850k platforms, respectively.

The main aim of ImmuMethy is to facilitate a global study of DNA methylation across samples of immune cells to identify differential methylation patterns and the underlying mechanisms. In addition to the storage of a huge number of methylation datasets, ImmuMethy allows several applications as follows. In addition, we performed an integrative analysis to support big data-driven knowledge discoveries.

#### A global view of methylation status and intensity

**Figure 3.**
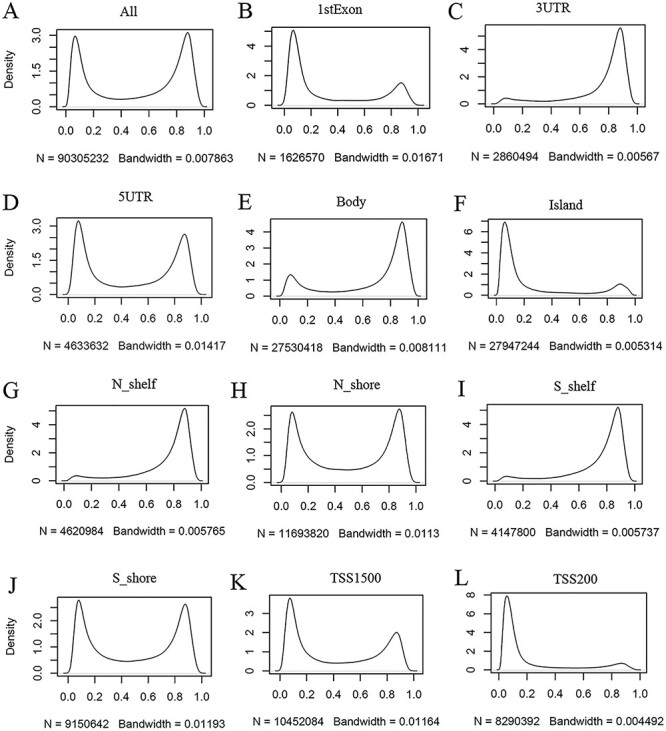
Kernel density estimation to show frequency distributions of beta values in different gene regions. The study dataset PB_CD4T_normal (normal peripheral blood CD4^+^ T cells) from the 450k platform is shown as an example. Kernel density estimation was analyzed using the R function “density” with default settings. “N” indicates the total number of methylation sites analyzed in each panel while “Bandwidth” represents a measure of how closely the kernel density matches the real distribution. Lower bandwidth is generally better. A total of 186 samples in the study dataset were uniformly processed, with each sample containing 485 512 cytosine sites. Therefore, a total of 90 305 232 sites in all samples were analyzed (A). These sites can be assigned to different gene regions based on their genomic loci (from panels B to L) in the associated genes, and then, the kernel density was estimated to show the beta value distribution of the cytosine sites in each gene region, as labeled in each panel. Gene regions explain as follows: 1stExon = the first exon; 3ʹUTR = Between the stop codon and poly A signal; 5ʹUTR = Within the 5ʹ UTR, between the TSS and the translational start site; Body = Between the translational start and stop codons, irrespective of the presence of introns, exons, TSS or promoters; Island  =  CGI; N shelf = 2–4 kb upstream (5ʹ) of CGI; N shore = 0–2 kb upstream (5ʹ) of CGI; S shelf = 2–4 kb downstream (3ʹ) of CGI; S shore = 0–2 kb downstream (3ʹ) of CGI; TSS1500 = 200–1500 bases upstream of the TSS; TSS200 = 0–200 bases upstream of the TSS.

ImmuMethy provides beta values and methylation profiles to illustrate methylation status and intensity. Although both uniformly processed and submitter-processed beta values are stored in ImmuMethy, methylation profiles, which are represented by r-beta curves, are processed only based on the uniformly processed data. An integrative analysis of the uniformly processed beta values reveals a generally bimodal distribution of beta values (Figure 3, first panel); however, regions of differential methylation status across different gene regions are observed. For example, in normal peripheral blood CD4^+^ T cells (PB_CD4T_normal), methylation sites located in CGIs, first exons and TSS200 (0–200 nt upstream of the TSS) generally show a hypomethylated status, whereas sites in gene bodies (the regions between the translational start codon and stop codon), 3ʹ UTRs and shelves (2–4 kb from CGIs) tend to be hypermethylated (Figure 3). Similar results were also observed in other immune cells (data not shown). This observation is consistent with current knowledge, as gene promoters have been reported to have low methylation levels (19, 20). ImmuMethy provides the methylation status and intensity of all sites in these gene regions and other sites that are currently without region annotations.

**Figure 4.**
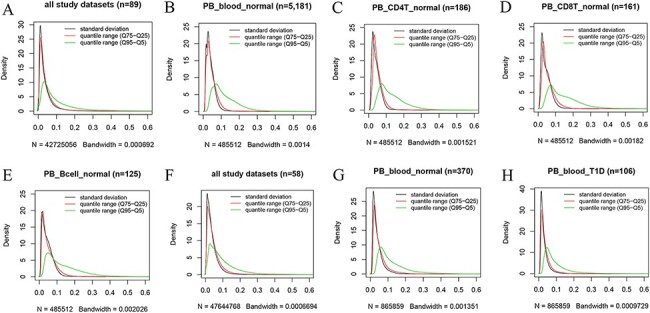
Kernel density estimation to show frequency distributions of standard deviation and quantile ranges. Figures A to E are from the 450k platform and indicate all study datasets (A), normal peripheral blood samples (B), normal peripheral CD4^+^ T cells (C), normal peripheral CD8^+^ T cells (D) and normal peripheral B cells (E). Figures F, G and H are from the 850k platform and indicate all study datasets (F), normal peripheral blood samples (B) and peripheral blood samples from individuals with type-1 diabetes.

A global view of beta values and methylation profiles allows easy identification of methylation intensity. In ImmuMethy, both the preprocessed and the uniformly processed beta values are aggregated for downloading. Although the preprocessed values derived from separate studies have not been normalized between samples, this only has a minor influence on methylation status evaluation at the global level because DNA methylation at each site is generally stable, with only minor changes seen in beta values in response to various experimental conditions (Figure 4, see below). For the methylation profile, the curve shape, such as width, height and peak position, can indicate the overall methylation intensity and its variability. Therefore, an overview of beta values or methylation profiles in ImmuMethy could provide quick insights into the methylation status and intensity of the queried sites and thereby enable a pre-evaluation of methylation levels before the experiment.

#### MPL analysis to show dynamic changes in DNA methylation

In this study, we used MPL to describe the variability in DNA methylation, that is, to what extent a cytosine modifies its methylation level under a series of conditions. MPL reflects a dynamic change in methylation modification under various conditions, such as cell/tissue sources and disease states. In the current study, MPL was evaluated based on the change of methylation levels at a single cytosine resolution. High plasticity indicates that it is common for DNA methylation to be highly variable under diverse circumstances, whereas low plasticity suggests relatively stable or small changes in methylation levels. Multiple methods, including quantile range (Q75–Q25), quantile range (Q95–Q5) and standard deviation as MPL scores, were used to quantitatively measure MPL for the uniformly processed data. Therefore, MPL levels of each site can be evaluated and compared according to these scores. Larger MPL scores indicate larger variability and higher plasticity. A cytosine site with extreme plasticity means it is highly or even fully methylated in some samples, whereas it shows very low or even no methylation in some other samples.

However, we found that DNA methylation generally shows low plasticity when the standard deviation and IQR are used to quantitatively measure MPL. For example, based on the distribution of the standard deviation of beta values in all 450k datasets (Figure 4A), there are ∼76%, ∼93% and ∼97% of all sites with standard deviations less than or equal to 0.05, 0.1 and 0.15, respectively. The median and mean standard deviation of all sites were only 0.02547 and 0.03873, respectively. Moreover, less than 2% of all sites had standard deviations larger than or equal to 0.2. The distribution of the “quantile range (Q75–Q25)” was quite similar to that of the standard deviation in all datasets (Figure 4A) and in specific datasets, such as normal peripheral blood cells (PB_blood_normal; Figure 4B), normal peripheral CD4^+^ T cells (PB_CD4T_normal; Figure 4C), normal peripheral CD8^+^ T cells (PB_CD8T_normal; Figure 4D) and normal peripheral B cells (PB_Bcell_normal; Figure 4E). For the 850k platform, similar results were also observed in all study datasets (Figure 4F) and in the specific datasets, such as “PB_blood_normal” (Figure 4G) and peripheral blood cells from individuals with type-1 diabetes (PB_blood_T1D; Figure 4H).

However, a quite different distribution pattern of “quantile range (Q95–Q5)” is observed in these datasets (Figure 4), suggesting much variation in beta value when this measure is used. For the 450k platform, there are ∼37%, ∼65% and ∼79% of all sites with quantile ranges (Q95–Q5) less than or equal to 0.05, 0.1 and 0.15, respectively. The median and mean of the “quantile range (Q95–Q5)” of all sites were 0.06793 and 0.10472, respectively. A similar result was observed for the 850k platform, and ∼34%, ∼64% and ∼80% of all sites had quantile ranges (Q95–Q5) less than or equal to 0.05, 0.1 and 0.15, respectively. Moreover, the median and mean of the quantile range (Q95–Q5) in all study datasets were 0.07293 and 0.09791, respectively.

In ImmuMethy, in addition to the above measures, the r-beta curve can directly illustrate MPL. High plasticity results in high quantile ranges and standard deviations of beta values and is generally indicated by a wide-shaped r-beta curve or a curve with multiple peaks. In contrast, low plasticity corresponds to low variability and is indicated by a narrow, single-peak r-beta curve.

#### Differential methylation analysis through methylation profile comparisons

ImmuMethy integrates different but functionally related studies into one study dataset; therefore, for differential methylation, it aims to illustrate large differences at a greater scale based on methylation distribution but does not focus on minor differences with very small beta value changes under specific conditions, as many of the source studies do. ImmuMethy comprises numerous blood cells subjected to various experimental conditions (including hundreds of disease states) and all main immune cell types, including B cells, CD4^+^ T cells, CD8^+^ T cells, natural killer (NK) cells, natural killer T (NKT) cells, macrophages, monocytes, dendritic cells and neutrophils, as well as their subsets (if methylome data are available). Therefore, differential methylation among different cell types or diseases can be analyzed by comparing their methylation profiles. A shift in the peak of the methylation profile in combination with the statistical tests can help to effectively identify differentially methylated sites.

ImmuMethy contains data from large numbers of control samples from normal individuals, such as normal blood, leukocytes, lymphocytes and peripheral blood mononuclear cells (PBMCs; Supplementary Tables S1 and S2). These abundant controls enable researchers to identify reliable differential methylation sites and facilitate the identification of disease-associated methylation markers. Therefore, re-evaluation of previously reported differentially methylated sites can be performed based on MPL, and novel differentially methylated sites can be identified.

**Figure 5.**
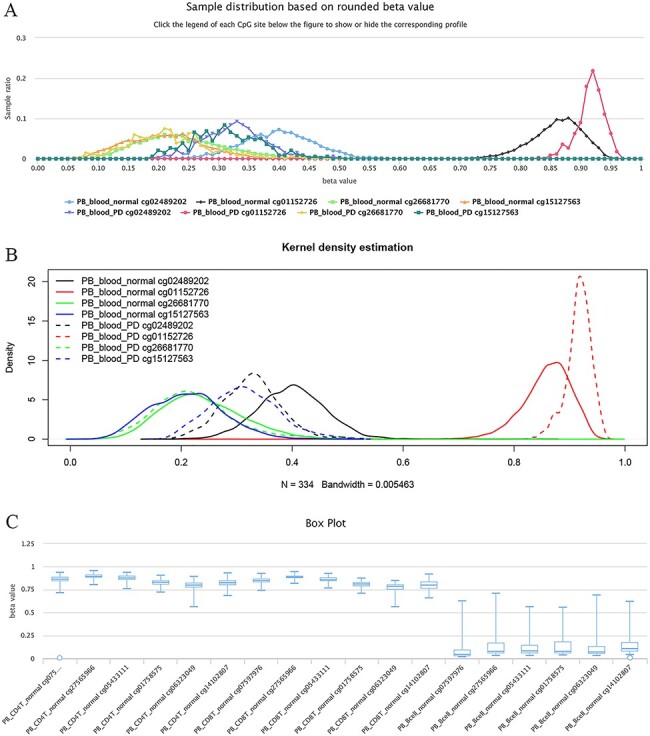
Examples of differential methylation sites. (A) Differentially methylated sites are illustrated by methylation profiles. In PD, site cg02489202 is hypomethylated, whereas cg01152726 is hypermethylated. However, there was no apparent differential methylation of cg26681770. Site cg15127563 shows an elevated methylation level in PD patients. The study datasets “PB_blood_normal” and “PB_blood_PD” indicate human peripheral blood samples from normal individuals (*n* = 5181) or individuals with PD (*n* = 334). The *y*-axis represents the sample ratio at an indicated beta value. For example, for cg01152726, there were 73 samples with a rounded beta value of 0.92, and the ratio was 73/334 ∼= 0.21856. Therefore, the accumulated square or ratio covered by each methylation profile curve is equal to 1. (B) Kernel density estimation to show frequency distributions of beta values of the sites in Figure A. Similar distributions of the same sites in Figures A and B can be observed, suggesting the feasibility and reliability of the r-beta curve to show the methylation profile. (C) Box plots showing differentially methylated sites in the CD19 gene in normal peripheral CD8^+^ T cells (PB_CD8T_normal, *n* = 161) and B cells (PB_Bcell_normal, *n* = 125). Datasets are from the 450k platform.

The site cg02489202 (located in the mitochondrial gene LARS2/leucyl-tRNA synthetase 2), for example, was previously reported as being the most significant Parkinson’s disease (PD)-related CpG (21). This site was also found to be hypomethylated in PD cases in ImmuMethy while cg01152726 (located in the gene LAMA3/laminin subunit alpha 3), which has been reported to be hypermethylated in patients (21), showed enriched methylation in ImmuMethy (Figure 5). However, some differential methylation sites identified in previous studies, such as cg26681770 (located in the gene PMEPA1/prostate transmembrane protein, androgen induced 1), do not show differential methylation in ImmuMethy (Figure 5). However, ImmuMethy revealed that cg15127563, which is found within the ITM2C gene (integral membrane protein 2C, also known as BRI3), is a novel differential methylation site with elevated methylation levels in PD patients (Figure 5). In a recent study, recombinant BRI3 protein was found to function as a molecular chaperone to inhibit amyloid formation and nonfibrillar protein aggregation (22). However, whether this CpG site is involved in the development of neurodegenerative diseases awaits further investigation.

Similarly, for the sites of cg07597976, cg27565966, cg05433111, cg01758575, cg06323049 and cg14102807 in the gene locus of CD19 (cluster of differentiation 19 molecules), which represents a specific marker gene in B cells, they are highly methylated in human CD4^+^ and CD8^+^ T cells, whereas lowly methylated in B cells (Figure 5C). More examples of significantly differentially methylated sites among normal human B cells, CD4^+^ T cells, CD8^+^ T cells, monocytes and neutrophils are listed in Supplementary Table S3.

ImmuMethy pays much attention to the most reliable differential methylation using large datasets based on beta value distribution. There was no overlap between any two r-beta curves, and the farther the peak distance was, the greater the difference in methylation intensity was.

However, because ∼62.29% (147/236) and ∼15.94% (11/69) of all study datasets for the 450k and 850k platforms, respectively, are not uniformly processed (Supplementary Table S1), only the preprocessed beta values but not methylation profiles can be analyzed for these study datasets. These cell types and diseases cannot be compared outside their own dataset, and ImmuMethy can only provide the preprocessed data. However, as ImmuMethy provides the source sample and study ID in the downloaded data file, it is possible to perform a precise comparison, particularly for small differential methylation between various conditions within the same study, i.e. studies with the same GSE ID in the downloaded file. It is an alternative option during the differential methylation analysis, particularly for sites without uniformly processed beta values (also see section “Discussion”).

#### Methylation tendency analysis based on beta value distribution

In the current study, we classified methylation sites into three categories based on the biased beta value distribution (see section “Materials and methods”). This classification is based on the sample population but not individual levels. The MTS and UTS reflect that the majority of the sites tend to be methylated and unmethylated, respectively, although unmethylated status for MTSs and methylated status for unmethylated tendency sites might also be observed in certain circumstances across samples. We did not use “hypermethylation” or “hypomethylation” to describe the tendencies in that these terms only represent an increase or decrease during DNA methylation comparison and do not consider beta value distribution in a sample population. However, for NTSs, it means that the cytosine sites are partially methylated with methylation levels adjacent to 0.5, or that similar sample proportions are observed to be, respectively, methylated and unmethylated across samples.

**Figure 6.**
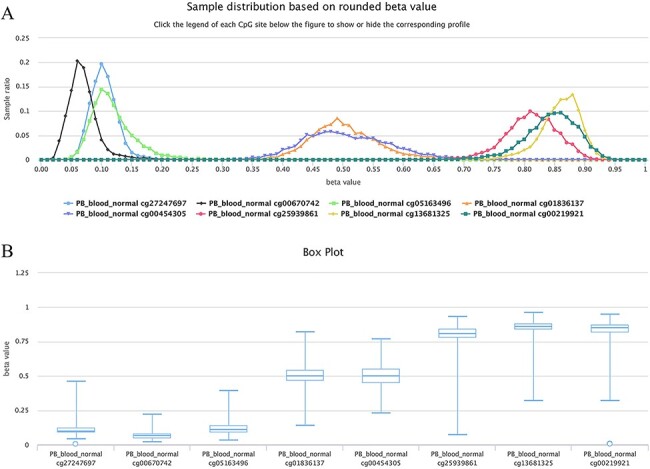
Examples showing methylation sites with differential methylation tendencies. (A) Methylation profiles showing differential methylation tendencies. (B) Box plots showing differential methylation tendencies of the same sites in Figure A. Datasets are from the 450k platform.

For example, as shown in Figure 6, all curves of the indicated sites contain the main peak. Although their overall methylation/unmethylation strengths are different, each site maintains a relatively stable methylation status. The sites including cg27247697, cg00670742 and cg05163496 in the CGI of the human gene CD8A belong to typical unmethylated tendency sites and tend to be unmethylated, which means that the methylated probe signals of these sites are often much lower than the unmethylated probe signals in the majority of normal peripheral blood samples. The sites including cg25939861 (S_Shelf), cg13681325 (S_Shelf) and cg00219921 (N_Shelf) of the same gene (CD8A) are typical MTSs and tend to be methylated, which means that the methylated probe signals of the sites are generally much higher than the unmethylated probe signals in the samples. A very small portion of sites had an unbiased methylation tendency across all samples. For example, for cg01836137 (located in the Island of gene INF2/inverted formin 2) and cg00454305 (located in the Island of gene UNKL/unk like zinc finger; Figure 6), the peak centers on a beta value of ∼0.5 in the situation, which means, although there is a variation to some extent, similar methylated and unmethylated probe signals were observed during array detection. The *P*-values were ∼0.37 and ∼0.08 for cg01836137 and cg00454305, respectively, during the tendency test in normal human peripheral blood cells (PB_blood_normal, *n* = 5181). Therefore, both of the sites belong to nonbiased methylation.

These three types of methylation sites can be easily discerned in ImmuMethy. More examples of MTSs, UTSs and NTSs are listed in Supplementary Table S4 (450k) and Supplementary Table S5 (850k). Differential methylation tendencies of a site under different conditions indicate increased MPL, and the conversion of methylation tendencies, particularly inversion from the methylation tendency to the unmethylation tendency, or vice versa, represents strong differential methylation. For example, the unmethylated tendencies of sites cg07597976, cg06323049, cg27565966, cg05433111, cg01758575 and cg14102807 in the gene locus of CD19 in B cells turn to methylation tendencies in T cells (Figure 5C). This highly differential methylation corresponds to the high expression of CD19 in B cells but no expression in T cells. More CpG sites with differential methylation tendencies are listed in Supplementary Table S6.

For the 450k platform based on our analysis, ∼54.67% (21 763 967/39 811 984), ∼42.92% (17 088 023/39 811 984) and only ∼2.41% (959 994/39 811 984) of all sites in the 82 study datasets with sample sizes larger than 3 were identified to be MTSs, UTSs and NTSs, respectively. This result means that ∼97.59% of the total cytosine sites present a significant tendency toward methylation or unmethylation. Furthermore, when we set a strict filter condition that all (100%) of the beta values of a site across samples were required to be larger than 0.5 for MTS or less than 0.5 for UTS, the result revealed that there were still ∼83.71% of all cytosine sites that showed strict tendentious distribution with ∼45.61% (18 159 128/39 811 984) of MTSs and ∼38.10% (15 170 118/39 811 984) of UTSs, respectively.

Similar results were also observed for the 850k platform. There were ∼62.30% (25 904 134/41 576 916), ∼33.51% (13 930 735/41 576 916) and ∼4.19% (1 742 047/41 576 916) of all sites that were identified to be MTSs, UTSs and NTSs, respectively. Under the above strict conditions, ∼56.80% (28 540 379/50 244 531) and ∼31.59% (15 874 047/50 244 531) of all sites presented methylation tendencies and unmethylation tendencies, respectively.

**Figure 7.**
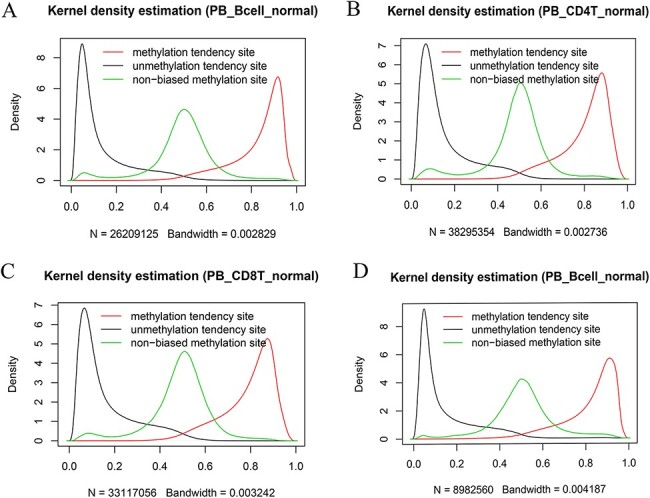
Kernel density estimation of beta values of the sites derived from the indicated categories. The figures show beta value distributions of the sites from MTSs, UTSs and nonbiased tendency sites in normal peripheral blood B cells (A, D), normal peripheral CD4^+^ T cells (B) and normal peripheral CD8^+^ T cells (C). Figures A, B and C are from the 450k platform, whereas D is from the 850k platform.

We also systematically examined the beta value distribution of the three types of tendency sites. As expected, MTS and UTS dominated to be methylated and unmethylated, respectively, whereas NTSs dominated to be partially methylated based on the traditional methylation site categories (see section “Discussion”). For example, among the MTSs (∼ 55.71% of the total sites) in the normal peripheral B cells of the 450k platform (Figure 7A), there were ∼70.85% of the sites with a mean larger than or equal to 0.8, whereas among the UTSs (∼43.19% of the total sites), there were ∼77.62% of the sites with a mean less than or equal to 0.2. Similar results were observed in normal peripheral CD4^+^ T cells (Figure 7B) and CD8^+^ T cells (Figure 7C) from the 450k platform and normal peripheral B cells (Figure 7D) from the 850k platform. Therefore, although most of the results are consistent, the current categories are not exactly the same as those of the traditional classification in that they consider MPL and data distribution.

An interesting finding was that similar results were also observed when the preprocessed beta values were used to calculate the methylation tendency. For example, among the 23 study datasets that had both preprocessed and uniformly processed data values, ∼97.33% (10 606 101/10 897 038) of all shared sites were identified to have the same methylation tendency. This result suggested that although the preprocessed beta values could not be used for precise differential methylation among different datasets, they could still be used for a general quick determination of methylation tendency.

Through ImmuMethy, methylation tendencies can be identified through a quick view of methylation profiles, which can be further checked based on the online downloaded beta values. A large sample size will make the methylation profile curves smoother and make it easier to discern distributional tendencies. The above results suggest highly biased tendencies of methylation distributions and further support a general limited variation in DNA methylation level in response to various experimental conditions. This means the absolute difference of methylation levels for differentially methylated sites is generally small; therefore, differential methylation should often occur in the sites with the same tendencies.

#### Sample subdivision or stratification based on highly plastic methylation sites

**Figure 8.**
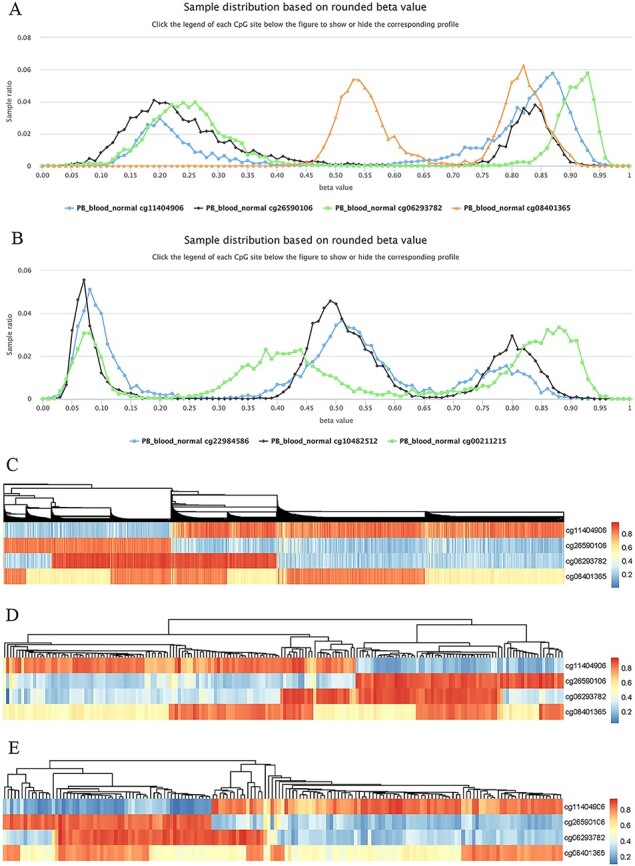
Examples to show split methylation sites. (A) Examples of split methylation sites with two main peaks in the methylation profiles. B. Examples of split methylation sites with three main peaks in the methylation profiles. Methylation profiles in A and B are from normal human peripheral blood samples (PB_blood_normal from 450k, *n* = 5181). C, D and E represent heatmaps to show sample subdivisions of normal human peripheral blood samples (C), normal human peripheral CD4^+^ T cell samples (D) and normal human peripheral CD8^+^ T cell samples (E) based on the split methylation sites in A, respectively. Color bars in C, D and E indicate DNA methylation from low (blue) to high (red) beta values.

Highly plastic methylation indicates highly differential methylation levels under different conditions. Although DNA methylation generally shows low plasticity, methylation sites with extremely high plasticity have been observed. For example, when considering “quantile range (Q75–Q25)” as a measure of MPL (see section “Discussion”), that is, MPL score, ∼0.06035% of sites (293/485 512) have an MPL score ≥ 0.5 and ∼0.8401% of sites (4079/485 512) have an MPL score ≥ 0.3 in normal peripheral blood CD4^+^ T cells. Interestingly, methylation profiles from highly plastic sites generally have multiple peaks; however, relatively stable methylation status can still be observed for each peak of the same site (Figure 8). For example, the sites cg11404906 (MPL score = 0.59; from the gene HLA-DRB1/major histocompatibility complex, class II, DR beta 1), cg26590106 (MPL score = 0.61; from the gene HLA-DRB1/major histocompatibility complex, class II, DR beta 1), cg06293782 (MPL score = 0.67; from the gene HLA-DQA2/major histocompatibility complex, class II, DQ alpha 2) and cg08401365 (MPL score = 0.28; from the gene IRAK1/interleukin 1 receptor-associated kinase 1) have two peaks in their profiles (Figure 8A), whereas the sites cg22984586 (MPL score = 0.47; from the gene CCR5/C-C motif chemokine receptor 5), cg10482512 (MPL score = 0.30; from the gene CCR6/C-C motif chemokine receptor 6) and cg00211215 (MPL score = 0.49; from the gene HLA-DRB1/major histocompatibility complex, class II, DR beta 1) have three peaks in their profiles (Figure 8B). This suggests that for such methylation sites, biased methylation tendencies occur in some samples while unmethylation tendencies are seen in other samples, and in some cases, nonbiased methylation tendencies can also be observed. Therefore, the aggregated distributions of beta values indicate that samples can be divided into different populations with differential methylation levels.

To systematically analyze the data distribution of beta values across samples for each site, we used the R package “fpc” with k-medoids algorithms to detect data homogeneity and further split beta values into the optimal clusters. As a result, ∼97% (450k) and  ∼ 99% (850k) of all sites in all datasets are homogeneous, which means that only one cluster for these sites was identified. This suggests that there are only a few cytosine sites that have obvious multiple peaks in their methylation profiles. For example, only 23 740 cytosine sites in human normal blood cells (PB_blood_normal) have multiple clusters (or groups) based on the beta value distribution. For the sites in Figure 8, the cluster number for cg11404906, cg26590106, cg06293782 and cg08401365 was correctly identified to be 2, which was the same as the peak number (Figure 8A), and the cluster number for cg22984586, cg10482512 and cg00211215 was 3, which was equal to the differential peak numbers in their methylation profiles (Figure 8B).

We use the term “split methylation” to describe the phenomenon whereby multiple methylation tendencies, or multiple aggregated tendentiousness of beta values, occur at a site within a study dataset. Therefore, split methylation represents a highly differentiated methylation status across samples. Split methylation sites can be identified from sites with high plasticity or with multiple clusters. It is typically indicated by two or three peaks in the methylation profile curve, meaning that samples can be subdivided into discrete groups based on the methylation status (Figure 8). For example, each site of cg11404906, cg26590106, cg06293782 and cg08401365 can separate human normal blood samples (PB_blood_normal) into two major groups with different methylation statuses (Figures 8A), whereas the sites cg22984586, cg10482512 and cg00211215 can divide the samples into three major groups with differential methylation levels (Figure 8B). Combinations of cytosine sites with split methylation can effectively make samples continuously stratified. For example, for the sites in Figure 8A, the combination of cg11404906 and cg26590106 makes human normal blood samples into two major groups, and the additional cg06293782 further makes the total samples into four major groups (Figure 8C). However, each group from the three-site combination could be further divided into two subgroups based on the methylation level of cg08401365 (Figure 8C). Similar results of sample grouping based on these sites have also been observed in human normal CD4^+^ T cells (Figure 8D), CD8^+^ T cells (Figure 8E), B cells and monocytes (data not shown). Therefore, the combination of split methylation sites can effectively stratify samples, and more sample subdivisions will be obtained when additional highly plastic cytosine sites are introduced.

Moreover, for highly plastic sites, it is possible to trace the corresponding experimental conditions based on the relevant source sample IDs so that the specific conditions associated with a specific cytosine site with a high or low methylation state can be determined. We identified all methylation sites with high plasticity, and in Supplementary Table S7, there are examples of methylation sites with MPL scores ≥ 0.5 based on quantile range (Q75–Q25) to obtain sufficient evidence or sample support.

The observation of split methylation prompted an investigation of the underlying mechanism. We found that genetic polymorphisms and alleles of CpG and single-base extension sites correlated with the majority of methylation sites with extremely high plasticity (Supplementary Table S7). For example, the sites cg11404906 (rs140028130/MAF = 0.028400; rs35497945/MAF = 0.011900; rs9269932/MAF = 0.297483; rs141975225/MAF = 0.086100; rs9269931/MAF = 0.400600; rs144660248/MAF = 0.039400), cg26590106 (rs9269762/MAF = 0.171882; rs12110879/MAF = 0.250000; rs112216378/MAF = 0.500000; rs9269761/MAF = 0.315789; rs9269760/MAF = 0.500000) and cg06293782 (rs2213565/MAF = 0.186397; rs148573253/MAF = 0.000661; rs1475411/MAF = 0.000567; rs1461151/MAF = 0.0002297) in Figure 8A contain multiple SNPs specific to their probes (Supplementary Table S7). Although these SNPs do not directly overlap the target CpG sites, the minor allele frequencies (MAFs) of some of them are very high (Supplementary Table S7). However, for site cg08401365 in Figure 8A, although no SNPs are observed in its probes, it is from the human X chromosome and represents sex-associated differential methylation sites. For example, the medians of beta values of cg08401365 were ∼0.54 and ∼0.82 in normal female (*n* = 2388) and male (*n* = 2306) blood samples, respectively. Therefore, two typical aggregated distributions of beta values at the site represent the underlying differential methylation in female and male populations (Figure 8A).

Similarly, the sites cg22984586 (rs2227010/MAF = 0.291018; rs186785168/MAF = 0.000500), cg10482512 (rs2021033/MAF = 0.466482) and cg00211215 (rs9269968/MAF = 0.457914; rs143441545/MAF = 0.055400; rs9269969/MAF = 0.218725; rs9269971/MAF = 0.461327) in Figure 8B also contain multiple SNPs, although these SNPs do not overlap the target cytosine site. Therefore, split methylation is closely related to genetic polymorphisms of array probes, and genetic variants in dataset samples could be evaluated to some extent based on the MPL seen in the profile curves.

Split methylation represents high plasticity and highly differentiated methylation. For different split methylation sites, the underlying mechanisms need further investigation because they represent real biological effects, whether it comes from genetic polymorphisms, gender differences or something else.

#### Analysis of TFs with predicted binding of methylated DNA

DNA methylation status can influence methylation-mediated protein–DNA interactions. In addition to the classical methyl-CpG binding domain (MBD) proteins, many TFs lacking an MBD, such as Kaiso and CCAAT/enhancer-binding protein-α (CEBPα), can interact with methylated DNA to mediate methylation-mediated biological processes (23). Therefore, we integrated information on methylated DNA–TF interactions based on the results from a recent study (18). Through the comparison of genomic coordinates, it was finally determined that the methylated DNA binding sites from 172 TFs completely overlapped with the cytosine sites in this study, involving a total of 46 519 and 65 116 CpGs for the 450k and 850k platforms, respectively (Supplementary Table S8). In addition, both high and low methylation binding sites of these TFs are included; therefore, it also facilitates a comparison between the observed methylation levels of the queried sites and the methylation levels of the binding sites in the original source of cell types.

The binding information of TFs also provides functional clues for the regulation of gene expression by genotypes. For example, the cytosine in the CpG site of cg00439656 (the 850k platform) from the gene ARHGAP30 (Rho GTPase activating protein 30) is a site of split methylation owing to SNPs with high MAFs in different populations (https://www.ncbi.nlm.nih.gov/snp/?term=rs12728349). As expected, the methylation profiles of cg00439656 in many study datasets in ImmuMethy, such as whole blood cells and PBMCs, comprise three typical peaks centered on approximately 0.89, 0.45 and 0.04 on the *x*-axis, representing methylated (genotype CC), partially methylated (genotype CT) and unmethylated (genotype TT) status, respectively. The high methylation status of the site is predicted to bind CTCF (CCCTC-binding factor) and RAD21 (RAD21 cohesin complex component) in H1-hESCs, although low methylation may also bind CTCF in HCT116 cells (Supplementary Table S8).

Nearly all 172 TFs can be expressed in the immune system based on RNA sequencing results in the DICE and ImmGen databases (24, 25). However, because methylation-dependent TF–DNA interactions may be cell type-specific (23) and the main sources of cell types for the TF–DNA interaction examination are not derived from immune cells, the application of ImmuMethy for predicted methylated DNA–TF interactions only implies functional clues for further experimental validation.

## Discussion

Although DNA methylation has been widely studied, a systematic analysis of the variability in DNA methylation using large sample datasets has not been reported. The abundant methylome data used in this study make it possible to quantitatively measure DNA MPL at a single cytosine resolution. ImmuMethy emphasizes exploiting the large sample size to reveal global DNA methylation patterns. With an increasing sample size, the methylation profile curve will become increasingly accurate and representative of broader populations. Moreover, in contrast to the majority of studies of differential methylation, this study addresses the limitations associated with identifying changes in differential DNA methylation. DNA methylation generally shows the minimal change in the absolute difference of methylation levels, even at differentially methylated sites. Although the inverted methylation tendency from methylation to unmethylation tendencies, or vice versa, represents strong differential methylation, the unique site is low in number. The inverted methylation tendencies generally occur among different datasets and still show limited ability to change methylation levels within the same dataset. Genetic changes in alleles are an effective way to change methylation tendencies, thus providing a way to understand the functional associations between genetic variation and genomic loci at the population level, such as gene regulation by TFs with differentiated methylation binding.

Although several database applications have been mentioned above, other potential functions of ImmuMethy are also implicated, such as the ability to evaluate probe quality based on methylation profiles, which will facilitate probe design and improve the efficiency of differential methylation detection. Moreover, because the sample information related to sex and age was also annotated in Supplementary Table S2, this makes it possible to identify gender- and age-associated differential methylation, such as aging clock sites. In addition, the methylation levels in the gene body and transcriptional regulatory regions, as well as the predicted TFs, are helpful to explain the underlying regulatory mechanism for differential gene expression.

Although the term “plasticity” commonly occurs in the literature, there is still a lack of research on how to quantify plasticity. In this study, we used MPL to describe the variability in DNA methylation. In statistics, there are many classical ways to describe the spread of data values, such as quartiles and standard deviations. Quartiles are much less affected by outliers and commonly expressed by an IQR, such as the “quantile range (Q75–Q25)” in this study. In contrast to the IQR, the standard deviation takes each value in a dataset into account and represents the average amount of variability. A high standard deviation indicates that values are generally far from the mean, whereas a low standard deviation represents that values are close to the mean. In this study, we found that beta values are frequently skewed with outliers, particularly for sites with split methylation or sites with high plasticity. In addition, we find that the distribution of “quantile range (Q75–Q25)” is more similar to standard deviation than that of “quantile range (Q95–Q5),” although both quantile ranges are closely correlated to standard deviation in all of the study datasets. Therefore, the “quantile range (Q75–Q25)” could represent a good choice as a quantitative measure of MPL, i.e. MPL score, particularly when enough samples are required to support MPL.

However, neither the “quantile range (Q75–Q25)” nor the “quantile range (Q95–Q5)” represent absolute plasticity, as the extreme difference or the range (the difference between the highest and lowest beta values of a site) does. For example, for the 450k platform, there are ∼75%, ∼50%, ∼37%, ∼28%, ∼17%, ∼11% and ∼7% of all sites with extreme differences larger than or equal to 0.05, 0.1, 0.15, 0.2, 0.3, 0.4 and 0.5, respectively, whereas for the 850k platform, there are ∼70%, ∼46%, ∼30%, ∼20%, ∼9%, ∼4% and ∼2% of all sites with extreme differences larger than or equal to 0.05, 0.1, 0.15, 0.2, 0.3, 0.4 and 0.5, respectively. These ratios are much higher than those obtained by other measures, such as “quantile range (Q75–Q25)” and “quantile range (Q95–Q5).” However, the extreme difference method is not able to distinguish an outlier from true values that are too large or too small and offers insufficient sample support. For example, for highly plastic sites with the same MPL score, the use of “quantile range (Q75–Q25)” will obtain more sample (50% of the data outside the range) support variability than the use of “quantile range (Q95–Q5)” (only 10% of the data outside the range); however, there are none of the data outside the range to support the plasticity when the range is used. Therefore, there are multiple ways to measure methylation variability, depending on how large the expected variability and the amounts of data samples to support the variability. Therefore, the values from the extreme difference are not currently integrated into ImmuMethy; however, users can still perform the analysis based on the downloaded data.

Methylation homeostasis is used to describe the finely tuned balance between de novo methylation, maintenance methylation, active demethylation and passive dilution of DNA methylation during cell replication (26). Cells need to actively methylate newly synthesized DNA over several cell divisions. In the current study, during quantitative measurement of MPL, DNA methylation was found to generally have the limitations of MPL and maintenance of a relatively stable DNA methylation tendency. Therefore, the current study further provides novel insight into methylation homeostasis.

In fact, several studies of differential DNA methylation have shown that the absolute difference in beta values of differential methylation sites is very small. For example, minor differences in beta values have been observed during fetal development (27), during aging of CD8^+^ T cells (28), and in race-associated methylation sites (29), along with many more examples from either array (30–34) or high-throughput sequencing (HTS) (35, 36). Moreover, studies have revealed that DNA methylation states in different tissues are highly positively correlated (37–39). This further supports the concept that under physiological conditions, DNA maintains epigenetic stability. The existence of highly plastic methylation sites not currently explained by genetic variation may suggest potential dysregulation of methylation homeostasis under some disease states. However, cell type-specific methylation should also be taken into account for highly plastic methylation profiles when multiple cell types are contained in a cell group, such as PBMCs, which comprise mainly T cells, B cells, NK cells and monocytes. This is because the heterogeneity of cell type-specific CpG methylation could potentially increase MPL or even lead to changes in methylation tendency at differential methylation sites. Therefore, it can also be inferred that even for the same cell type, purity during cell isolation may also lead to a change in MPL.

In high-throughput experiments, batch effects, which represent nonbiological variations related to experimental factors, such as laboratory conditions, reagent lots and personnel differences, will lead to increased variability and decreased power to detect true biological signals (40). Improved experimental design (such as proper randomization) and the use of statistical solutions for batch correction effectively reduce the batch effect; however, the effectiveness of statistical techniques such as ComBat truly depends on a proper experimental design, and even so, batch effects may not be completely removed (40–42). This suggests that not all potential sources of batch effects could be successfully identified and corrected.

However, the batch effect is not the main concern for current research motivation. In the current integrative analysis, a large number of samples were collected from various studies; therefore, randomized samples may minimize positional effects, which are from unbalanced sample placement in different physical positions within the same chips (41). Moreover, the raw data in the IDAT file format were uniformly processed to try to reduce the impact of study batches. Batch correction generally aims to resolve the unwanted increased variations, particularly during the differential analysis. However, the differential methylation analysis is only one of the database applications. The current findings have shown general DNA methylation with limited but not increased variability. Batch effects only affect a subset of probes, and the absolute difference in methylation level was found to be small between various batches (42). Although batch effects may still exist in the uniformly processed data, we paid less attention to small differences in a methylation level, which could be observed due to technical variations than to true biological differences. Therefore, in addition to statistical significance, an absolute difference in beta value and differentiated region methylation are helpful to identify true biological differences.

During the differential methylation analysis, probes associated with frequent SNPs, which may result in unwanted variation, are generally excluded during the data analysis (41, 42). However, these probes related to SNPs are not removed in the database, because in the current situation the sites with SNPs can be used to evaluate the methylation status affected by genotypes and may have real biological consequences. In addition, based on methylation profiles of large data samples, sample stratification and outlier samples can be also evaluated irrespective of SNPs. This helps to acquire prior knowledge about methylation status, intensity and tendency, which facilitates further experiments to identify true biological differences.

The current single sample normalization has great benefit for analyzing large datasets because data can be processed separately so that new analysis can be updated with data accumulation independently of the previous analysis. This study focuses on an overview of data, discovery of molecular rules and providing biological insights. Therefore, the preprocessed beta values were also integrated into ImmuMethy for a quick global view of data to obtain a preliminary understanding of methylation status, intensity and tendency. In addition, these preprocessed data can help users trace their datasets of interest in the GEO and ArrayExpress databases to make a more accurate analysis.

During the analysis, the SWAN algorithm was used to adjust the design for Infinium I and Infinium II probes. We also investigated the differences in beta values before and after SWAN and found that the difference was generally less than 0.1, suggesting a minor change in absolute methylation levels after correction. In this study, submitter processed data include both normalized and nonnormalized data, but most of the data are normalized by submitters using different methods. Although it is highly heterogeneous among these data, the absolute difference in methylation level is actually small based on our analysis. For example, there were ∼4100 samples for which both the uniformly normalized and preprocessed beta values were provided; however, only the former was deposited in ImmuMethy. We compared the difference between these beta values and found that the difference in absolute beta values was larger than 0.1 for only ∼5% of sites in each sample. Therefore, the method of analysis should only affect the interpretation of methylation values for a small proportion of cytosine sites. This suggests that for the majority of cytosine sites, the preprocessed beta values from a mix of different studies can still be used for a quick evaluation of methylation status, intensity and tendency with ImmuMethy and the heterogeneity of the preprocessed data is not the main concern for an overview of data.

However, as mentioned above, the preprocessed data cannot be directly used for comparison among different datasets. For precise comparisons and when more differentially methylated sites with minor absolute differences are expected, the analysis can be performed within the same datasets, such as the same GEO series. However, the large difference in a methylation level provides the easier interpretation of biological effects, and the significant small difference may still result from the undiscovered batch effect. Therefore, in addition to statistical significance, it is also important to use absolute differences in methylation levels to identify more reliable differential sites.

Arrays and NGS are dominant tools in the epigenome-wide association study field. WGBS has the greatest advantage in the quantitative determination of the methylation states of all CpG sites in the human genome, but it is very expensive. Reduced representation bisulfite sequencing is more cost effective, but datasets based on this protocol still await accumulation. All current data are array-based, as the sample sizes are much larger than those of HTS. However, studies have revealed that the methylation values determined by arrays and HTS platforms are highly correlated (43–45), with the correlation coefficients reaching over 0.98 in some cases (44). This suggests that the hypothesis of MPL and homeostasis presented in this study should remain valid, even when enough HTS data have been accumulated and analyzed. In fact, using WGBS data of immune cells from BLUEPRINT (http://dcc.blueprint-epigenome.eu/#/files), we performed a preliminary analysis, and the results also support the current conclusions about limited DNA MPL (data not shown).

High reproducibility at the 450k CpG sites in the 850k platform has been observed (14). However, microarray technologies do not identify methylation signals specific to positive and negative DNA strands separately, nor do they distinguish allele-specific signals. In addition, nonspecific hybridization signals for some probes may occur. Therefore, genome-wide methylome sequencing of large quantities of samples can further improve our understanding of DNA methylation and its tendency. We aim to produce this database in a timely update and include datasets from HTS platforms in the future.

Traditionally, an individual CpG site is classified as fully methylated (close to 1), unmethylated (close to 0) and partially methylated (close to 0.5) based on a variable cut-off of DNA methylation levels. However, different studies use different cut-offs to define methylation status. For example, a CpG site is classified as methylated and unmethylated based on DNA methylation levels higher than 80% and less than 20%, respectively, and others (20% to 80%) are classified as partially methylated (46, 47). In some studies, the partially methylated sites were further classified into three categories: intermediate between partially methylated and methylated (60–80%), partially methylated (40–60%) and intermediate between unmethylated and partially methylated (20–40%) (48). Another study considered methylation levels less than 60% as low methylation and higher than 80% as high methylation (23). In addition, methylation levels of ≥90% or ≤10% CpG sites were considered fully methylated (100%) or unmethylated (0%), respectively (49). In a recent study based on the hidden Markov model, individual CpG sites were identified to be full-methylated site (FMS), middle-methylated site (MMS) and unmethylated site (UMS) based on cell type-specific cut-offs (50). For CpG sites, the cut-off separating UMSs from MMSs is approximately 0.2 in both GM12878 and H1-hESC cells, whereas the cut-off separating MMSs from FMSs is approximately 0.75 and 0.88 in GM12878 and H1-hESC cells, respectively (50). Therefore, various thresholds were used to define methylation status in different studies.

In this study, we divided methylation sites into three categories: MTSs, UTSs and NTSs. The classification aims to reflect the tendency of methylation level distribution at the population but not individual levels. Therefore, although the categories are closely associated with differentiated methylation status, the classification does not determine whether a site is fully methylated or fully unmethylated under a specific condition. This is because of the dynamic characteristics of DNA methylation and the various functional states of single cells contained in bulk samples. Therefore, even for MTSs, an unmethylated status may also be observed under certain conditions at some cytosine sites. In contrast, for unmethylated sites, methylation may occur under some other conditions. Bulk samples comprise multiple single cells with various methylation statuses, even for the same cell types; therefore, the methylation tendency reflects an overall probability of being methylated or unmethylated.

In contrast to a previous study (50), which predicts methylation status according to the CpG site context under a specific condition, the current results do not reflect the mutual associations among cytosine sites. It is interesting and worth further investigating their spatial correlation in the next step. Although the current categories are from a different perspective and not the same as those mentioned above, the majority of unmethylated and methylated sites belong to unmethylated and methylated sites (Figure 7), respectively, based on the traditional definition thresholds. However, the current tendency detection is based on statistical tests only from two intervals, although other intervals of interest may also be selected, depending on different research purposes. However, it is necessary to properly understand the underlying biological and nonbiological meanings, particularly for the sites with peaks adjacent to 0.5 and the sites with split methylation in methylation profiles.

MPL reflects the dynamic change in methylation levels in response to conditions. Low plasticity indicates a small change in methylation levels across samples, whereas high plasticity indicates a large change in methylation levels. Inverted methylation tendencies, such as from unmethylation to methylation, or vice versa, indicate higher plasticity than that of differentiated methylation within the same tendency sites. Plasticity is tightly associated with layering, and high plasticity provides the chance of sample division or stratification through component deconvolution. These divisions, as well as their further subdivisions, lead to sample heterogeneity, which implies dissimilarity and diversity. Therefore, plasticity explains the underlying mechanism of heterogeneity. Therefore, the plasticity analysis facilitates “marker” evaluation, which implies homogeneity and stability (although these qualifications are relative). For example, DNA methylation markers imply that they are lowly plastic.

In our previous studies, we performed the quantitative measurement of gene plasticity, which was referred to as expressional gene plasticity (51), and potential applications based on plasticity analysis were implicated, such as marker gene evaluation (51), novel immune cell subpopulation identification (52) and internal phenotype analysis according to the correlated and anticorrelated expressional relationships (53). The current study focuses on gene plasticity at the DNA methylation level, i.e. methylational gene plasticity. Therefore, gene plasticity can be expressed at multiple levels, such as the levels of transcriptome and methylome. Other levels of gene plasticity at proteome, exome and other types of epigenomes and so on, are under further study. In contrast to expressional plasticitomes (52), methylational plasticitomes comprise all plastic methylation sites, which will inform functional studies of epigenetic modifications involved in the regulation of the corresponding methylation sites and further biological consequences. All these contribute to the study of plasticitomics.

## Supplementary Material

baac020_Supp

## Data Availability

All data can be available upon request.
